# Insights into the Allosteric Regulation of Human Hsp90 Revealed by NMR Spectroscopy

**DOI:** 10.3390/biom15010037

**Published:** 2024-12-30

**Authors:** Tjaša Goričan, Simona Golič Grdadolnik

**Affiliations:** Laboratory for Molecular Structural Dynamics, Theory Department, National Institute of Chemistry, Hajdrihova 19, p.p. 660, SI-1001 Ljubljana, Slovenia; tjasa.gorican@ki.si

**Keywords:** Hsp90, NMR spectroscopy, allosteric regulation, allosteric modulators

## Abstract

Human heat shock protein 90 (Hsp90) is one of the most important chaperones that play a role in the late stages of protein folding. Errors in the process of the chaperone cycle can lead to diseases such as cancer and neurodegenerative diseases. Therefore, the activity of Hsp90 must be carefully regulated. One of the possibilities is allosteric regulation by its natural allosteric modulators—nucleotides, co-chaperones and client proteins—and synthetic small-molecule allosteric modulators, such as those targeting the middle domain or the C-terminal domain (CTD) of Hsp90. Since no experimentally determined structure of a small-molecule allosteric modulator bound to the CTD of human Hsp90 has yet been obtained, the challenge for a structure-based design of allosteric modulators remains. Solution nuclear magnetic resonance (NMR) spectroscopy could be utilized to overcome these problems. The main aim of this review article is to discuss how solution NMR techniques, especially protein-based, and the advanced isotope labeling of proteins have been used to investigate the allosteric regulation of the cytosolic isoforms of human Hsp90 with allosteric modulators. This article provides the basis for planning future NMR experiments, with the aim of gaining insights into allosteric sites and the mechanisms of allosteric regulation.

## 1. Introduction

**Figure 1:**
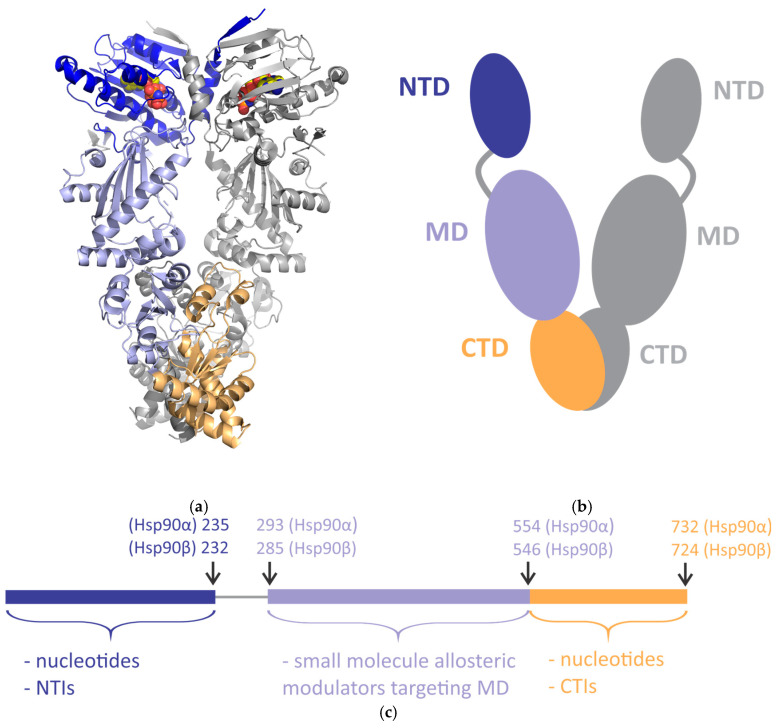
The domain structure of human cytosolic heat shock protein 90 (Hsp90). The domains in one of the protomers are shown in different colors (the N-terminal domain (NTD) in blue, the middle domain (MD) in light purple and the C-terminal domain (CTD) in light orange). The domains in the other protomer and charged linkers are shown in gray. (**a**) Cryogenic electron microscopy (cryo-EM) structure of human Hsp90β with the adenosine triphosphate (ATP) analog phosphoaminophosphonic acid-adenylate ester (ANP) bound to the “canonical” ATP-binding site in the NTD (protein data bank (PDB) code 8EOB [18]). The protein structure is shown as a cartoon and ANP as spheres. (**b**,**c**) Schematic representation of the domain structure of the dimeric human Hsp90 (**b**) and one of its protomers (**c**). (**c**) Nucleotides can bind to the NTD and the CTD. Synthetic small-molecule inhibitors can target the NTD (NTIs), and synthetic small-molecule allosteric modulators can bind to the MD or the CTD (CTIs). The arrows indicate the first or last residue in each domain of Hsp90α and Hsp90β [14].

In the human body, proteins must be correctly folded in order to maintain their tertiary or quaternary structures, which is essential for their function. Human heat shock protein 90 (Hsp90) is one of the main chaperones that acts in the late folding stages and enables the proper folding, processing and maturation (activation [1,2] and stabilization [3]) of its client proteins [4,5]. The domain structures of human Hsp90 isoforms are similar, as each identical protomer of a dimeric protein contains an N-terminal domain (NTD), a middle domain (MD) and a C-terminal domain (CTD) (Figure 1) [4]. In addition, the human cytosolic isoforms Hsp90α and Hsp90β have a sequence identity of approximately 85% and contain a charged linker between the NTD and the MD in each protomer [4]. All three domains in each protomer of human Hsp90 contain sites for interactions with co-chaperones and client proteins [6,7,8,9,10,11,12,13,14,15,16]. Binding sites for nucleotides are located in the active site of the NTD and the allosteric site of the CTD [17].

During the process of the chaperone cycle, Hsp90 undergoes large conformational changes essential for its function [4,5]. According to the general model of the Hsp90 chaperone cycle, adenosine triphosphate (ATP) binds to the NTD of cytosolic Hsp90 in a C-terminally dimerized state [4]. The binding of co-chaperones to Hsp90 supports the recruitment, transfer and processing of client proteins [4]. This leads to conformational changes that result in the N-terminal dimerization of Hsp90 and the hydrolysis of ATP, allowing the chaperone cycle to begin again [4]. Errors in this process can lead to diseases such as cancer and neurodegenerative diseases [4,19]. Therefore, the activity of Hsp90 has to be precisely regulated under physiological conditions [4]. One of the possibilities could be allosteric regulation by nucleotides, co-chaperones and client proteins, which bind to allosteric sites and thereby inhibit (decrease) or activate (increase) ATPase activity in the active site of the NTD of Hsp90 [9,20,21,22]. This slows down or accelerates the chaperone cycle. In particular, the binding of the co-chaperones Hsp70/Hsp90-organizing protein (Hop) and p23 inhibits the ATPase [4]. On the other hand, the co-chaperone activator of the Hsp90 ATPase protein 1 (Aha1) increases the ATPase rate and, subsequently, accelerates the chaperone cycle [4]. In addition, the binding of co-chaperones regulates the binding of client proteins [4]. Furthermore, Hsp90 is a potential target for the treatment of cancer, neurological disorders and infectious diseases [19]. According to the binding sites, synthetic small-molecule orthosteric N-terminal inhibitors (NTIs) targeting the NTD and synthetic small-molecule allosteric modulators targeting the MD or the CTD (CTIs) have been developed (Figure 1) [19,23,24]. Of these, only NTIs have so far reached clinical trials [24]. However, the Hsp90 inhibitor pimitespib has successfully reached clinical practice in Japan for the treatment of advanced gastrointestinal stromal tumor, confirming Hsp90 as a clinically relevant target for cancer treatment [19,25]. Non-selective NTIs were unsuccessful, as they caused off-target effects and the induction of the heat shock response (HSR), leading to increased toxicity [19,23]. With selective CTIs, these problems can be mitigated [19,23]. However, the lack of an experimentally determined structure of CTI bound to human Hsp90 is one of the main challenges for the rational design and development of optimized analogs [23]. Alternatively, nuclear magnetic resonance (NMR) techniques can be used to investigate the binding of CTIs to Hsp90 in solution [26].

The aim and scope of this review article is to discuss recent advances in the chemical shift assignment and knowledge of interactions with allosteric modulators and subsequent conformational changes of human cytosolic Hsp90 isoforms obtained by NMR techniques. The purpose of this work was to collect information on how NMR techniques in solution can be used to investigate the mechanisms of the allosteric regulation of human Hsp90. This could provide the basis for planning future NMR experiments, with the aim of mapping the interactions and allosteric conformational changes and gaining information on molecular mechanisms. This is important because it will help to overcome the current problems in structure-based design and the development of optimized allosteric modulators with therapeutic potential.

## 2. Allosteric Regulation of Human Hsp90 Investigated by NMR Spectroscopy

**Figure 2:**
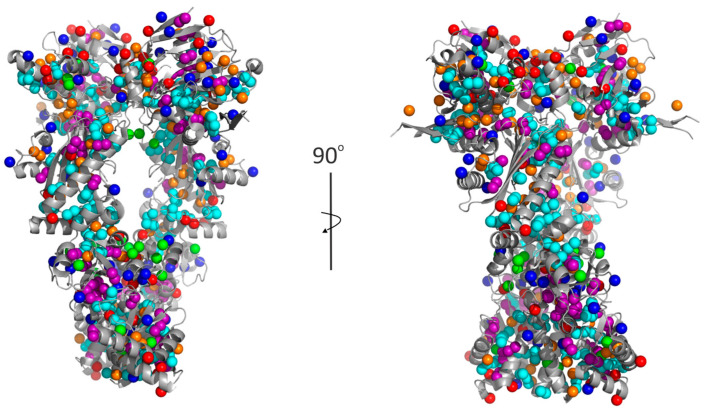
Cryo-EM structure of human Hsp90β, showing that the methyl groups excellently cover the protein. The protein is shown as a cartoon, with the methyl groups of residues, Ala (red), Ile (orange), Leu (light blue), Met (light green), Thr (blue) and Val (purple), shown as spheres (PDB code 8EOB [18]). For clarity, ANP is not shown.

Human Hsp90 activity can be regulated by allosteric modulators that bind to allosteric sites in the CTD, the MD or interface regions, which are spatially distant from the active sites in the NTD [20,26,27]. The binding of allosteric modulators and the subsequent conformational changes of the protein can be detected in the NMR spectrum as perturbed resonances of specific residues [9,20,21,22,26,27]. The chemical shift assignment of full-length human Hsp90 provides a basis for mapping allosteric binding sites and conformational changes by NMR techniques. Assigning the chemical shifts of full-length human Hsp90 in solution is a challenging task due to the limitations of NMR techniques related to the high molecular weight of the protein [5]. Since full-length human Hsp90 has a molecular weight of approximately 170 kDa and disordered regions, perdeuteration and advanced isotope labeling schemes, such as methyl-specific labeling, are required to obtain well-resolved resonances in NMR spectra recorded with specially developed NMR techniques [5,9,14,20,21,22,28,29,30,31,32] based on transverse relaxation-optimized spectroscopy (TROSY) [33]. The specific labeling of selected nuclei reduces the overlap of the crowded NMR spectra of large proteins, while the TROSY technique suppresses transverse relaxation, which reduces the loss of signal intensities and line broadening, thus improving spectral sensitivity and resolution [34]. The cross-relaxation enhanced polarization transfer (CRINEPT) technique [35] enables a further significant increase in sensitivity compared to TROSY [34]. In addition, the deuteration of proteins suppresses dipole–dipole relaxation, leading to reduced line broadening and improved spectral sensitivity, as deuterium has a lower magnetic dipole moment compared to the proton [5]. Moreover, methyl-specific labeling enables the higher sensitivity and resolution of TROSY spectra [36,37]. This is because methyl groups are not rotationally constrained and three protons contribute to the signal. In addition, the total number of signals is lower because not all residues are labeled but only specific residues bearing methyl groups (Ala, Ile, Leu, Met, Thr and Val) [5]. According to the protein structure, the methyl groups excellently cover each domain of Hsp90 (Figure 2) [22].

The resonance assignments of full-length Hsp90 have been accelerated by transferring the available assignments from the isolated NTD and MD domains [9,20,22,38]. However, the chemical shift assignment of the isolated CTD of human Hsp90 was not successful, as the protein signals were very broad, probably due to aggregation [14]. According to the literature, all specifically ^13^CH_3_-labeled Ala-β, Ile-δ_1_, Leu-δ_2_, Met-ε, Thr-γ and Val-γ_2_, (AIL^pro-S^MTV^pro-S^) methyl group resonances and most backbone resonances of the human Hsp90 NTD have been assigned (Table 1) [39,40]. The long residence time resorcinol type inhibitor (ligand) 5-[4-(2-Fluoro-phenyl)- 5-oxo-4,5-dihydro-1H-[1,2,4]triazol-3-yl]-*N*-furan-2-ylmethyl-2,4-dihydroxy-*N*-methyl-benzamide bound to the Hsp90α NTD enabled the backbone assignment of the segment covering the ATP-binding site [39]. Assignments were also reported for the backbone and the Ile-δ_1_ methyl group resonances of the isolated MD and the monomeric two-domain construct NTD + MD of human Hsp90α [14]. All Ile-δ_1_ resonances and most backbone resonances of the human Hsp90 MD have been assigned [14]. In addition, the Ile-δ_1_ resonances of the truncated homodimeric Hsp90α construct (2 × (1–732)) with part of the charged linker between the NTD and the MD (241–268) removed (Hsp90Δ) have been assigned [14]. In the case of the full-length human Hsp90β, all Ile-δ_1_ methyl group resonances and 35% of the Met-ε resonances have been assigned so far [20,22]. For the human Hsp90β CTD, all Ile-δ_1_ resonances and one Met-ε methyl group resonance have been assigned [20,22]. Chemical shift assignments of Ile methyl groups have enabled the investigation of the allosteric regulation of full-length human Hsp90 by allosteric modulators, the synthetic small molecules **KU-32**, **KU-596** and **SOMCL-16-171**, the nucleotides ATP and its analogs, the co-chaperones p23, Aha1 and the Hop, and the client protein mineralocorticoid receptor (MR)-LBD [9,20,21,22,26]. Nevertheless, there is still a lack of knowledge about their molecular mechanisms [9,20,21,22,26,27].

### 2.1. Allosteric Regulation of Human Hsp90 with CTIs

**Figure 3:**
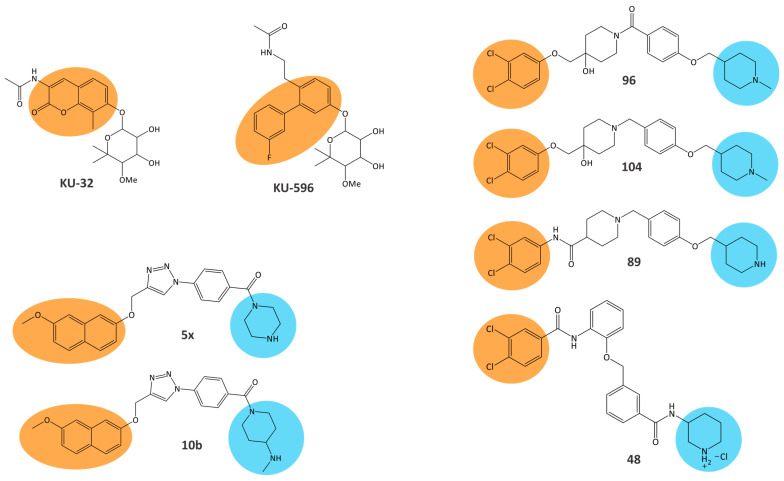
Structures of CTIs of human Hsp90 investigated by saturation transfer difference nuclear magnetic resonance (STD NMR) [26,49,50,51]. The orange-colored circles mark the CTI moieties that received the highest degrees of saturation and were therefore in the closest contact with Hsp90. Blue circles mark the moieties that contributed the least to the binding of CTIs.

The allosteric regulation of human Hsp90α with the ATP analog AMP-PNP and two CTIs, the novobiocin analogs **KU-32** and **KU-596** (Figure 3), was investigated using methyl TROSY [26]. The chemical shift perturbations (CSPs) of Ile-δ_1_ resonances from each domain were detected in the spectra of full-length Hsp90α with specifically ^13^CH_3_-labeled Ile-δ_1_ methyl groups in the absence and presence of AMP-PNP or the individual analogs **KU-32** and **KU-596** [26]. Some of these Ile-δ_1_ resonances originated from the vicinity of interfaces between different adjacent domains (I214, I361 and I494) or the NTD dimer interface (I110), according to the structure of full-length yeast Hsp90 in closed conformation (protein data bank (PDB) code 2CG9) [6,26]. The shift of two Ile-δ_1_ resonances from the CTD was observed, but their assignments are not known [26]. Since no changes were observed in the ^1^H-^15^N TROSY-heteronuclear single quantum coherence (HSQC) spectrum after the addition of **KU-596** to the ^15^N-labeled isolated NTD of Hsp90α in terms of signal shift or broadening, it was confirmed that this ligand does not bind to the “canonical” ATP-binding site [26]. The addition of AMP-PNP to full-length Hsp90α resulted in CSP of the same Ile-δ_1_ resonances from the CTD, as in the case of **KU-32** and **KU-596**, without affecting other signals from the CTD [26]. Therefore, it was shown that ATP, novobiocin, **KU-32** and **KU-596** bind to the “cryptic” ATP-binding site in the CTD of Hsp90α [17,26,43,44]. Moreover, after the addition of individual ligands **KU-32** and **KU-596**, CSP of the same Ile-δ_1_ resonances from the NTD and the MD were detected, as in the case of AMP-PNP [26]. Therefore, AMP-PNP, **KU-32** and **KU-596** induce long-range (global) allosteric conformational changes, which may be propagated to the NTD of Hsp90α via a common pathway [26].

Using a pyruvate kinase/lactic dehydrogenase (PK/LDH)-coupled assay, it was shown that the binding of **KU-32** to human Hsp90 increased the rate of ATP hydrolysis (ATPase activity) [45]. In addition, the binding affinity of adenosine diphosphate (ADP) for the Hsp90–**KU-32** complex was lower than that for Hsp90 alone [45]. On the other hand, the binding affinity of ATP was higher for the Hsp90–**KU-32**–ADP complex than for Hsp90 alone [45]. This indicates that Hsp90 in complex with **KU-32** favors the release of ADP and subsequent ATP binding [45]. Furthermore, molecular dynamics simulations of the Hsp90–**KU-32**–ATP complex revealed an initial movement of the Hsp90 protomers toward each other [45]. This is consistent with the hypothesis that the global conformational changes of Hsp90 upon binding with **KU-32** lead to the formation of a “partially closed” intermediate, which enables increased ATPase activity [45].

It is worth mentioning that in the discovery and optimization of new chemical classes of CTIs, ligand-based NMR techniques, saturation transfer difference NMR [46] and transferred nuclear Overhauser effect spectroscopy (trNOESY) [47] can also be very useful for determining the binding contributions of the different ligand moieties and the conformations of the bound ligands [26,48,49,50,51,52]. These techniques are not limited by the size of the protein and require only small amounts of non-labeled protein, but the information collected is mainly limited to the properties of the bound ligand [53]. The binding of designed CTIs—the analogs of novobiocin (with noviose and acetamide moieties) [26], 1,4-disubstituted triazoles [49] and compounds with 3,4-dichlorophenyl moieties [50,51]—was investigated by STD NMR (Figure 3). The CTI moieties with the highest and lowest degrees of saturation were determined using group epitope mapping analysis of STD amplification factors [54]. The moieties with the highest degree of saturation were in the closest contact with the Hsp90 binding site contributing the most to the binding of CTIs (Figure 3) and can be used as the basis for derivatization. The moieties with the lowest degree of saturation, which contribute the least to binding, can be replaced in the optimization process. Although the possible determination of the protein binding sites of 1,4-disubstituted triazoles and compounds with 3,4-dichlorophenyl moieties by STD competition experiments [55] was hampered by the low solubility of these CTIs in buffers suitable for proteins [49,51], the binding mode of selected derivatives, **48**, **89**, **96** and **104,** in the CTD was proposed according to the determined conformation of the bound derivatives using trNOESY and molecular dynamics simulations of their complexes with Hsp90 [50,51]. Furthermore, the time-resolved fluorescence energy transfer (TR-FRET) technique was used to confirm the binding of these derivatives to the CTD of human Hsp90 [49,50,51].

The CTIs of human Hsp90 investigated by ligand-based methods were also tested for their therapeutic potential [26,49,50,51]. Compounds **KU-32** and **KU-596** were found to have neuroprotective effects and therapeutic potential for the treatment of diabetic peripheral neuropathy [26,56]. Moreover, **KU-32** was able to improve diabetic sensory neuropathy in mice with diabetes [57,58]. On the other hand, 1,4-disubstituted triazoles and compounds with 3,4-dichlorophenyl moieties inhibited the proliferation of the Ewing sarcoma cell line SK-N-MC (**5x**, **10b** and **48**) and various breast cancer cell lines: two hormone-dependent cell lines, MCF-7 (**5x**, **10b**, **89**, **96** and **104**) and T47D (**89**, **96** and **104**), a HER2-overexpressing cell line, SKBr3 (**89**, **96** and **104**), and a triple negative breast cancer (TNBC) cell line, MDA-MB-231 (**89**, **96** and **104**) [49,50,51]. Compounds **3** and **5x** caused cell-cycle arrest in the cancer cell lines SK-N-MC (**3**) and MCF-7 (**5x**) [49,51]. In addition, compounds **3**, **5x**, **89** and **104** induced apoptosis in cancer cell lines SK-N-MC (**3** and **5x**), MCF-7 (**5x**) and MDA-MB-231 (**89** and **104**) [49,50,51]. These compounds decreased the levels of the oncogenic Hsp90 client protein estrogen receptor α (ERα) in MCF-7 (**5x, 89** and **104**) and c-rapidly accelerated fibrosarcoma (c-Raf) in SK-N-MC (**3**) [49,50,51]. Moreover, they did not induce HSR, and they slowed tumor growth in xenograft models with SK-N-MC tumor (**3** and **5x**) or TNBC tumor (**89** and **104**) [49,50,51]. It has thus been shown that these allosteric CTIs could be used for further optimization with potential for the development of cancer therapeutics [49,50], and it is worth investigating their allosteric regulation using protein-based techniques like methyl TROSY.

### 2.2. Allosteric Regulation of Human Hsp90 with the Synthetic Small-Molecule Allosteric Modulator Targeting the MD

The allosteric regulation of human Hsp90α with the compound SOMCL-16-171 was investigated by domain-specific isotope labeling [27]. For this purpose, the Hsp90α fragment with the ^2^H-, ^15^N-labeled NTD and the unlabeled MD was used [27]. The resonances of many residues in the NTD, including L80, R201 and K209, were duplicated in the ^1^H-^15^N HSQC spectrum, which indicates that these residues exist in two conformations in solution [27]. Upon addition of the compound SOMCL-16-171, one of the two resonances of each duplicate disappeared [27]. This indicates that residues K209, R201 and L80 undergo conformational shifts in the presence of the compound [27]. Furthermore, no significant spectral changes were observed in the ^1^H-^15^N HSQC spectrum with an ^2^H-, ^15^N-, ^13^C-labeled isolated NTD in the presence and absence of the single compounds SOMCL-16-171 and SOMCL-16-175 [27]. This indicates that the binding of the compound SOMCL-16-171 induces a long-range allosteric effect on the NTD of Hsp90α [27].

The binding of compounds SOMCL-16-171 and SOMCL-16-175 to the MD of Hsp90α was demonstrated by ^1^H-^15^N HSQC using the ^2^H-, ^15^N-, ^13^C-labeled isolated MD in the presence and absence of each compound [27]. In particular, residues with significantly perturbed and attenuated chemical shifts were identified in four fragments spanning a loop region, D372-G387, and two α-helices, K443-E451 and I522-E535, which are spatially close to each other [27]. They are also spatially close to the highly flexible loop region F349-N360, which undergoes slow conformational exchange in solution [27]. Therefore, the backbone resonances in this region (F349-N360) are not present in the NMR spectra and could not be assigned [27]. By the mutagenesis study, it was confirmed that the binding site for the compounds spans two loop regions, F349-N360 and D372-G387, and one α-helix, K443-E451 [27]. There were almost no interactions between SOMCL-16-175 and MD F349A or D350A, and there were less significant CSPs of the L382 and K443 resonances when SOMCL-16-175 was added to MD L382A or MD K443E compared to the wild-type (wt) MD [27]. However, when SOMCL-16-175 was added to MD Y528A, the CSP of the Y528 resonance was similar to that of the wt MD [27]. Therefore, the α-helix spanning I522-E535 may not be involved in the binding of the SOMCL-16-175 compound [27]. The observed CSP in the α-helix region spanning I522-E535 most likely indicates conformational changes caused by the allosteric modifiers SOMCL-16-171 and SOMCL-16-175 [27]. There is no experimentally determined structure of SOMCL-16-171 or SOMCL-16-175 bound to human Hsp90α. Therefore, the allosteric effect could not be related to the conformation of human Hsp90α with the bound compound. However, using an ATP hydrolysis assay, it was shown that the binding of SOMCL-16-171 and SOMCL-16-175 to Hsp90α accelerated its ATPase activity in vitro [27]. According to the results of proteomic analysis and immunoblot data, SOMCL-16-175 destabilizes the client proteins of Hsp90α [27]. These contradictory results indicate that ATPase activity does not fully correlate with the activity of the chaperone cycle [27].

### 2.3. Allosteric Regulation of Human Hsp90 with Co-Chaperones and Nucleotides

#### 2.3.1. Allosteric Regulation of Hsp90β with p23 and ATP

The allosteric regulation of human Hsp90β with its co-chaperone p23 in the presence of ATP or its analogs was investigated using methyl TROSY [9]. The CSP of the Ile-δ_1_ resonances from the NTD of full-length human Hsp90β with specifically ^13^CH_3_-labeled Ile-δ_1_ methyl groups was detected in the presence of a non-hydrolysable analog (AMP-PNP or ATPγS) or using an ATP-regenerating system [9]. These shifts originated mainly from the ATP-binding pocket and its lid (I53, I75, I90 and I122) and the helical region required for the dimerization of the NTD (I20, I27 and I28) (Table 2) [9]. Similar shifts were also observed with the isolated NTD upon the binding of AMP-PNP [9]. However, split Ile-δ_1_ resonance signals in addition to CSP for I90 were only observed in the spectra of the full-length protein (Table 2) [9]. Therefore, it was shown that ATP induces conformational changes in the NTD of Hsp90β, particularly in the ATP-lid and proximal regions that determine the dimerization of the NTD [9]. No significant CSP of the Ile-δ_1_ resonances from the MD and the CTD of full-length Hsp90β were observed in the presence of a non-hydrolysable analog (AMP-PNP or ATPγS) or using an ATP-regenerating system [9]. It was concluded that ATP does not induce global conformational changes in all the domains of human Hsp90β but only local changes in the NTD [9]. The results obtained with methyl TROSY are consistent with the proposed model obtained by the crosslinking reaction and electron microscopy [59]. According to this model, apo Hsp90 exists in three conformational states that are in dynamic equilibrium [59]. It is predominantly present in an open state but also in closed and compact states [59]. ATP binds to both NTDs of Hsp90 and shifts the equilibrium to the closed state [9,59]. However, only very minor change occurred in human Hsp90 compared to Hsp90s from *E. coli* and yeast [59].

**Figure 4:**
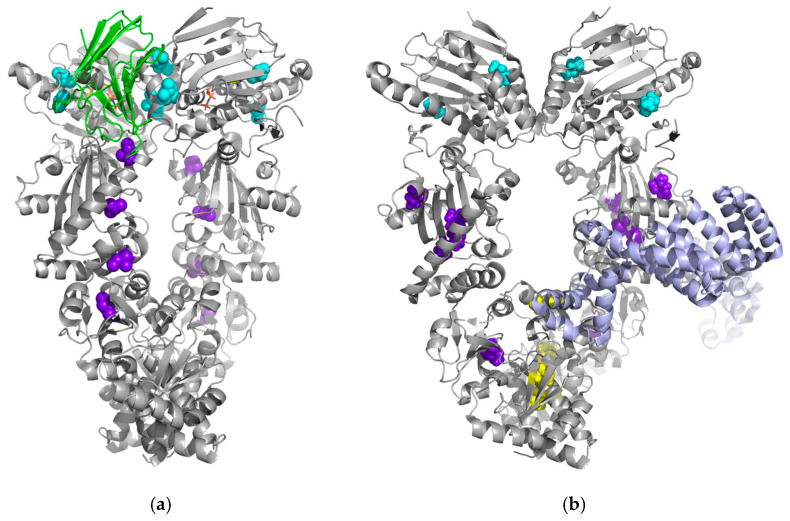
Cryo-EM structures of human Hsp90α showing the Ile residues near and far from the binding sites of p23 (**a**) and Hop (**b**). The residues with Ile-δ_1_ resonances that were perturbed after the binding of p23 in the presence of ATP or Hop to Hsp90β are shown as spheres (the NTD in cyan, the MD in purple and the CTD in yellow). The protein structures are shown as a cartoon (Hsp90α in gray, p23 in green and Hop in purple). ANP is shown as sticks. PDB codes 7L7J (**a**) and 7KW7 (**b**).

The CSP of the Ile-δ_1_ resonances from both the NTD and the MD after the addition of p23 to full-length human Hsp90β with specifically ^13^CH_3_-labeled Ile-δ_1_ methyl groups in the presence of ATP (using an ATP-regenerating system) was detected by methyl TROSY [9]. The CSP from the NTD originated from the lid over the ATP-binding pocket and the underlying layer (I20, I27, I28 and I122) as well as from the adenine end of the ATP-binding pocket, which is distant from the lid (I90) (Table 2) [9]. In addition, CSPs were detected over large parts of the MD, and most of them were distant from the binding site of p23 (I369, I440 and I482), as shown by the structures of the yeast Hsp90–p23 complex, determined by X-ray crystallography (PDB code 2CG9), and the human Hsp90α–p23 complex, determined by cryogenic electron microscopy (cryo-EM) (PDB code 7L7J) (Figure 4) [6,9,60]. The latter indicates that p23 induces long-range conformational changes in the MD and provides insights into the allosteric regulation of Hsp90β by p23 [9]. The co-chaperone p23 binds to the NTD and connects both protomers of human Hsp90 [9]. In addition, the complex is stabilized by its interaction with the MD [9]. The binding of p23 to human Hsp90 further stabilizes the closed state, as shown by the crosslinking reaction and electron microscopy [59]. It also restricts the NTD–MD rotation required for ATPase activity and thus inhibits the hydrolysis of ATP [59]. The two atomic models of Hsp90 in the complex with p23 obtained by X-ray and cryo-EM are similar [60]. However, in contrast to the crystal structure of the yeast complex, the structure of the human complex indicates that a single p23 molecule binds to the human Hsp90 dimer (Figure 4) [60]. By native mass spectrometry, it was shown that Hsp90 and p23 form a complex in a 2∶2 ratio [9]. The contradictory results of cryo-EM and native mass spectrometry can be explained by the results of methyl TROSY, as the splitting of a subset of peaks may reflect an asymmetry in an Hsp90–p23 complex [9,60].

#### 2.3.2. Allosteric Regulation of Hsp90β with Aha1

**Figure 5:**
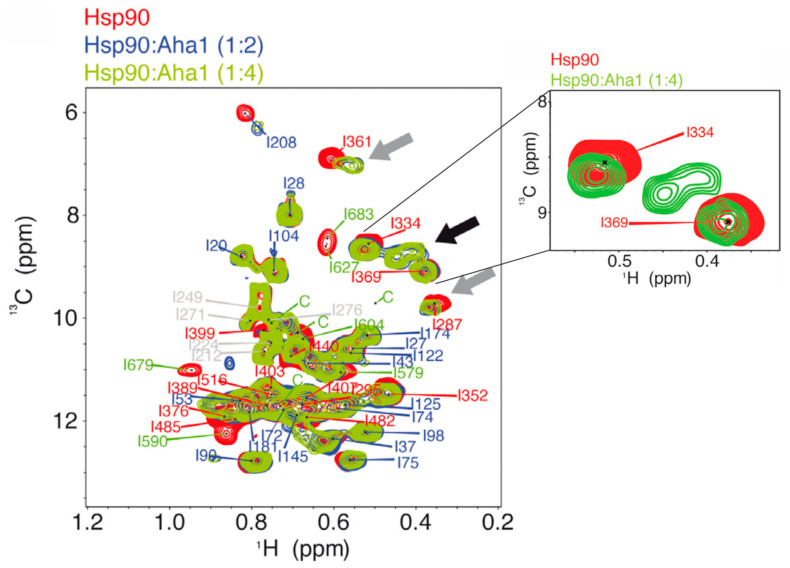
The methyl-TROSY NMR spectrum of full-length human Hsp90β with specifically ^13^CH_3_-labeled Ile-δ_1_ methyl groups in the absence and presence of Aha1, as shown by J. Oroz et al. [20]. The addition of Aha1 led to strong changes in the NMR spectrum, including chemical shift perturbations (CSPs) and cross-peak broadening (shown as gray arrows) and the appearance of new cross-peaks (shown as black arrow). Cross-peaks corresponding to four unassigned residues in the CTD are marked by C.

Using methyl TROSY, the allosteric regulation of human Hsp90β with its co-chaperone Aha1 was investigated [20]. Strong changes regarding CSP, a CSP of decreased magnitude (cross-peak broadening) and the appearance of new cross-peaks upon the addition of Aha1 to full-length human Hsp90β with specifically ^13^CH_3_-labeled Ile-δ_1_ methyl groups in the absence of a nucleotide were observed (Figure 5) [20]. Changes in the Ile-δ_1_ resonances from the NTD (I20, I27, I28, I37, I98, I122 and I125) and the MD (I361 and I389) were detected (Table 2) [20]. The broadening of the cross-peaks was the strongest in the case of the Ile residues at the interface between the MD and the CTD and at the NTD dimer interface involved in dimer closure [20]. Both interfaces are rearranged during allosteric changes in yeast Hsp90 [61]. Furthermore, the binding of Aha1 leads to a conformational rearrangement of Hsp90β from an open to a partially closed conformation [38,42]. Also, the cross-peak broadening of the Ile-δ_1_ resonances from the MD regions of the binding site for Aha1 have been observed according to the crystal structure of the yeast complex between the Hsp90 MD and Aha1 (PDB codes 1USV and 1USU [61,62]) [20]. Therefore, changes in Ile-δ_1_ resonances were detected for the residues involved in the interaction with Aha1 and allosteric changes in Hsp90β [20]. To distinguish between these residues, the monomeric construct of Hsp90β was prepared with the NTD and MD regions, which are the most important regions for the interaction with Aha1 (NTD + MD) [20,38,61,62]. Therefore, Aha1 can bind to the NTD + MD construct, but it cannot induce allosteric changes leading to dimer closure [20]. Using methyl TROSY, it was shown that the Ile-δ_1_ resonances for I20, I27, I37, I75 and I208, located at the interface between the NTD and the MD and at the NTD dimer interface, were broader in the case of the full-length Hsp90β bound to Aha1, reflecting allosteric changes leading to dimer closure (Table 2) [20]. On the other hand, the Ile-δ1 resonances for I53, I72, I74, I122, I125, I145 and I181, which form the region in the NTD, were broader in the case of the NTD + MD construct, reflecting the interaction between the Hsp90β NTD and Aha1 in the process of binding [20].

By methyl TROSY, changes in Ile-δ_1_ resonances were detected for I20, I27, I28, I37, I98, I122, I125, I361 and I389 upon the binding of ADP to full-length Hsp90β with specifically ^13^CH_3_-labeled Ile-δ_1_ methyl groups (Table 2) [20]. Similar changes were observed upon the binding of ATP or Aha1 in the absence of nucleotides [20]. Therefore, it was hypothesized that the binding of Aha1 to Hsp90β could induce allosteric conformational changes, involved in NTD dimerization, toward an intermediate state [20]. The latter could enable ATP exchange and energetically facilitate the additional changes required for ATP binding, trapping and hydrolysis, which would increase the activity of Hsp90β [20,61,63,64,65]. This hypothesis is consistent with previous studies showing that Aha1 is important for accelerating the ATPase activity of human Hsp90 [63].

#### 2.3.3. Allosteric Regulation of Hsp90β with Hop

The co-chaperone Hop was used to investigate the allosteric regulation of human Hsp90β by methyl TROSY [21]. The CSP, splitting and broadening of signals from each domain of full-length human Hsp90β with specifically ^13^CH_3_-labeled Ile-δ_1_ methyl groups were observed at increasing concentrations of Hop [21]. Signal splitting indicates the presence of two states of Hsp90β [21]. At low Hop concentrations (1:0.2 molar ratio of Hsp90:Hop), signal changes were detected mainly for residues of the CTD (I485, I590, I604 and I679), which is consistent with previous findings that Hop can interact with the C-terminal MEEVD motif of yeast Hsp90 (Table 2) [21,66]. In the case of a molar ratio of 1:0.5 of Hsp90:Hop, signal splitting was observed for residues of the NTD and the MD (I20, I75, I287, I334, I376 and I407) [21]. To demonstrate the Ile residues near and far from the binding site for Hop on human Hsp90, we used the cryo-EM structure of human Hsp90α in complex with Hop, Hsp70 and a client protein, the glucocorticoid receptor (GR), at a 3.6 Å resolution (Figure 4) [67]. The structure reveals that in the presence of Hop, Hsp70 and GR, Hsp90 adopts a “semi-closed” conformation, with a rotated NTD that has not yet reached the fully closed state [67]. This conformation is important for the binding and activation of clients and for ATP hydrolysis [67]. In the case of a 1:1 molar ratio of Hsp90:Hop, the signal intensities corresponding to one of the Hsp90β states decreased to approximately 30% [21]. Furthermore, it was demonstrated by isothermal titration calorimetry (ITC) and size-exclusion chromatography with multi-angle static light scattering (SEC-MALS) that one Hop molecule binds the dimeric Hsp90α (2:1 stoichiometry), and it is assumed that the complex is stable in this ratio [68,69,70]. Using these results from NMR, ITC and SEC-MALS, it was demonstrated that the binding of one Hop molecule to the CTD of dimeric Hsp90β induces an allosteric conformational change between both Hsp90β monomers [21]. Most Ile-δ_1_ resonances from the dimer interface remained unperturbed upon the binding of Hop according to the closed structure of human Hsp90β [11,21]. This is in contrast to Aha1, which induces a closed conformation of Hsp90β [20,21]. Using size-exclusion chromatography and dynamic light scattering, it was shown that Hop preferentially interacts with the open conformation of Hsp90β [21]. This is consistent with the observation that perturbed residues within the MD and CTD interface are not accessible for interaction with Hop when Hsp90 is in its closed state [21]. Therefore, the binding of Hop to the open conformation does not induce the transition of Hsp90β to the closed state, which is consistent with the extended conformation observed by SEC-SAXS experiments [21]. By preventing the closing of Hsp90, Hop acts as an inhibitor of the ATPase and prevents the hydrolysis of ATP [4].

The perturbed Ile-δ_1_ resonances correspond to two regions of the CTD and the MD that face each other in the open model of Hsp90β [21,38]. One of the reasons for the latter could be that Hop binds to CTD and induces the conformational rearrangement of Hsp90β toward a V-shaped conformation with a V-shaped interface between the MD and the CTD [21]. Moreover, the V-shaped conformation of Hsp90α stabilized by Hop was structurally characterized by cryo-EM at a resolution of 15 Å [68]. Furthermore, the NMR signal perturbations from NTD residues are consistent with the cryo-EM structure of the Hsp90α–Hop complex, where the NTD of Hsp90α was found to be rotated 90° toward the ATP-bound state [68,69]. These data obtained by NMR and cryo-EM support the formation of a V-shaped conformation of the Hsp90–Hop complex and may indicate that the NMR signal perturbations from the NTD residues are due to conformational changes and not the binding of Hop to the NTD [21,67]. However, the V-shaped conformation is inconsistent with a model of the Hsp90–Hop complex based on negative strain electron microscopy, in which Hsp90α was found to be in a semi-closed, ADP-bound state [70]. By using a truncated Hop construct, Hop112a, which is sterically unable to reach the NTD when bound to the MEEVD motif in the CTD, the possibility of the direct binding of Hop to the NTD was ruled out [21,66]. After increasing the concentration of Hop112a, signal perturbations were detected in methyl TROSY spectra for the same Hsp90β residues at the V-shaped interface between the MD and the CTD, as well as in the NTD, as in the case of the full-length Hop [21,68,69]. Therefore, the binding of Hop to the CTD induces conformational changes in the NTD and stabilizes Hsp90β in a V-shaped conformation (Table 2) [21]. This conformation supports the transfer of the client proteins from Hsp70 and their interaction with Hsp90 [4].

### 2.4. Allosteric Regulation of Human Hsp90 with the Client Protein

In addition to nucleotides and co-chaperones, the client protein MR-LBD was used to investigate the allosteric regulation of human Hsp90β by methyl TROSY [22]. After the addition of the MR-LBD, the CSP of the Ile-δ_1_ and Met-ε resonances in each domain of full-length human Hsp90β with specifically ^13^CH_3_-labeled Ile-δ_1_ and Met-ε were detected [22]. By intermolecular paramagnetic relaxation enhancement (PRE) experiments with spin-labeled clients, MR-LBD contact regions were revealed, and therefore, the effects of the interactions and conformational changes were distinguished [22]. The highest CSP values were determined for the CTD, which is probably due to allosteric effects [22]. However, to our knowledge, there are no data on the connection between the allosteric conformational changes and the conformation of human Hsp90 in complex with MR-LBD. Furthermore, there is no information on how MR-LBD regulates human Hsp90 (its ATPase activity). CSP can reveal the allosteric regulation of proteins at the residue resolution [71]. However, the lack of information on protein structure and function (enzyme activity) poses a challenge for the interpretation of the results obtained with methyl TROSY in terms of allosteric regulation. Investigations into protein function by biochemical assays of the wt protein and their mutant forms with mutations close to the potential allosteric site are essential for the confirmation of the allosteric effects [72]. In addition, these assays can provide information on how the binding of allosteric modulators to the allosteric sites affects catalytic activity and the binding of other ligands to other sites on the protein [72]. In this way, the importance of allosteric sites in the allosteric regulation of proteins can be determined [72]. This knowledge may be useful for the design and development of potential allosteric therapeutics that target the allosteric site and, subsequently, regulate protein function [72].

## 3. Discussion

According to the PDB, several protein structures of full-length human Hsp90 in complex with proteins or small molecules have been determined by cryo-EM. However, there is no experimentally determined structure of a small-molecule allosteric modulator bound to the CTD of full-length human Hsp90. On the other hand, some structures of small-molecule allosteric modulators bound to the allosteric sites of other proteins have been determined by cryo-EM [73,74,75]. They have provided the possibility to visualize the bound small molecules in the allosteric sites of proteins and to determine their binding modes. However, it is generally difficult to obtain structural information about proteins at the atomic level using cryo-EM [72]. Furthermore, it is still not possible to obtain atomic information for highly dynamic, flexible protein regions [72]. Therefore, it is usually necessary to use prior knowledge, such as higher resolution structures from X-ray crystallography, to fit low molecular weight ligands into cryo-EM maps [73,76]. However, the recently developed technique of time-resolved cryo-EM enables various transient intermediate states occurring on the millisecond, and even microsecond, timescale to be obtained, which provides a static view of dynamic processes (insights into protein dynamics) [77]. Unfortunately, it cannot capture the continuous dynamics of proteins, and there are still challenges with the resolution of transient states [77]. In addition, investigating the dynamic processes at millisecond timescales remains difficult due to sample heterogeneity and radiation damage during data acquisition [77]. Cryo-EM technology is currently undergoing rapid improvement and development [73,75]. It is expected that many structures of compounds bound to proteins will be obtained by cryo-EM in the future [75]. This gives cryo-EM a potential value for the structure-based design of optimized CTIs and for gaining insights into their mechanisms of action.

One of the main advantages of NMR spectroscopy compared to cryo-EM for the design of allosteric modulators is that it enables the structure of proteins in solution to be obtained under physiological conditions and dynamic processes (protein dynamics) to be investigated at a higher resolution (at the atomic level) and over a wide range of timescales (from picoseconds to seconds) [72,73,77]. In addition, NMR makes it possible to characterize the dynamics of flexible protein regions and their interactions at the atomic level over a wide range of timescales [72]. Another advantage is the possibility of identifying multiple conformational states simultaneously [72]. Furthermore, relaxation dispersion NMR enables the detection and characterization of the low-populated, transient excited, intermediate states of biomolecules at high-resolution [77]. NMR also provides insights into transient interactions [72]. NMR could complement cryo-EM in the design of allosteric CTIs by providing atomic-level information on low-populated, transient states, protein dynamics and conformational changes that fills the gap between the static structures (conformations) obtained by cryo-EM. In addition, NMR could complement cryo-EM to improve the resolution of the protein structures of human Hsp90 with bound CTIs. This could enable a more detailed understanding of the structure and molecular mechanisms [74] of CTIs bound to Hsp90 and the identification of new allosteric sites, which would contribute to the structure-based design [74,75] of optimized CTIs.

Studies on the allosteric regulation of human Hsp90α using protein-based NMR showed that the binding of synthetic small-molecule allosteric modulators to the CTD or the MD induced long-range allosteric effects on the NTD. However, depending on the binding site, they caused different changes in resonances in the methyl TROSY spectra. This could indicate that they act via a different mechanism. These synthetic small-molecule allosteric modulators increase the ATPase activity of human Hsp90. However, allosteric modulators targeting the MD have been shown to slow down the chaperone cycle [27]. Therefore, it appears that they have additional functions that affect the chaperone cycle but have not yet been explored. Furthermore, experimentally determined structures of the complexes of these small-molecule allosteric modulators bound to human Hsp90 are lacking. However, by a computational method, it was shown that the binding of one of the CTIs leads to the movement of the protomers toward each other, which is consistent with the partially closed conformation of human Hsp90 [45].

According to methyl TROSY, the binding of the individual co-chaperones p23 (in the presence of ATP) and Hop, similar to synthetic small-molecule allosteric modulators, also induced long-range allosteric conformational changes in human Hsp90β. This is in contrast to the binding of ATP, which only caused local changes. The splitting of the resonances (the appearance of new cross-peaks) could indicate the presence of multiple conformational states of human Hsp90 with bound ATP, p23 (in the presence of ATP), Aha1 or Hop. This is consistent with the proposed model suggesting the equilibrium between the open, closed and compact states of human Hsp90 with bound ATP [59]. In particular, it might reflect a dimeric molecule of human Hsp90 with one or two bound molecules of p23 (different stoichiometries) [9,60]. Moreover, by determining the ratios of the two states, it was shown that the binding of Hop induced allosteric effects between both Hsp90β monomers [21]. The closed state with restricted NTD–MD rotation and a V-shaped conformation of Hsp90β formed upon the binding of the individual co-chaperones p23 and Hop may be associated with their function in inhibiting ATPase activity. Furthermore, the binding of Aha1 results in a similar partially closed conformation of human Hsp90β, as in the case of a synthetic small-molecule allosteric modulator targeting the NTD of human Hsp90α. This conformation may also be associated with their function in activating ATPase activity. Overall, many conformational states of human Hsp90 with bound nucleotides and co-chaperones have been discovered using NMR and other experimental techniques. In addition, apo Hsp90 exists in three conformational states that are in dynamic equilibrium according to the proposed model obtained by the crosslinking reaction and electron microscopy [59]. It is predominantly present in an open state, but also in closed and compact states [59]. However, no conformational state of full-length human Hsp90 with bound small-molecule allosteric modulators in the CTD has been detected by experimental methods.

Comparing the perturbed Ile-δ_1_ resonances in the methyl TROSY spectra, most differences in the MD of human Hsp90β are observed in the case of p23 (in the presence of ATP) compared to ADP, Aha1 and Hop and in the case of Hop compared to p23 (in the presence of ATP), ADP and Aha1 (Table 2). This could be due to different mechanisms of action of p23 and Hop. However, it is difficult to compare these results, as they were not obtained with the same buffer compositions. Differences in salt concentrations and, consequently, in ionic strength may influence the allosteric effects [78]. CSPs are very sensitive to structural or dynamical changes and enable the investigation of allosteric interactions and conformational changes at residue resolution [71]. In addition to CSP analysis and PRE experiments, allosteric regulation can also be investigated by other techniques, such as relaxation dispersion experiments, which provide insights into protein dynamics (exchange dynamics between interconverting states) over a range of timescales and at the atomic level [71,72,77]. To our knowledge, the dynamics of allosteric regulation of human Hsp90 have not yet been investigated by NMR relaxation experiments. In the future, relaxation experiments such as Carr–Purcell–Meiboom–Gill (CPMG) and chemical exchange saturation transfer (CEST) could be used for investigating the allosteric regulation [72] of human Hsp90. Methyl-based CPMG and CEST NMR experiments could provide information on the chemical exchange, the conformational dynamics on the intermediate timescale and the low-populated excited states [71,72] of human Hsp90.

## 4. Conclusions and Outlook

To date, no experimentally determined structure of a small-molecule allosteric modulator bound to full-length human Hsp90 CTD has been obtained. However, the assignments of all Ile-δ_1_ methyl group resonances of full-length human Hsp90β could allow the mapping of allosteric sites and conformational changes and the investigation of the dynamics of protein complexes in the process of allosteric regulation in the future. Therefore, NMR techniques may be important for the rational design and development of allosteric modulators of human Hsp90 as potential cancer therapeutics. Compared to orthosteric inhibitors, allosteric modulators have the potential to be more selective for any cytosolic isoform of human Hsp90. This is because the NTD of these isoforms is evolutionarily better conserved than other domains. The selective inhibition of human Hsp90 could allow for fewer side effects in cancer treatment. Moreover, allosteric therapeutics have the potential to be safer than orthosteric therapeutics, as they do not completely block protein activity but regulate it [79]. Key challenges in investigating the allosteric regulation of Hsp90 by NMR remain the high molecular weight and flexibility of Hsp90. Recently, protein-observed fluorine NMR has emerged as a promising technique for investigating the molecular mechanisms of Hsp90, including ligand binding, conformational changes and changes in dynamics [80,81,82,83,84,85,86]. The methyl-specific labeling of other residues in combination with site-directed mutagenesis could reveal additional allosteric sites of human Hsp90. This could allow a more detailed investigation of the mechanisms of allosteric regulation by NMR spectroscopy.

## Figures and Tables

**Table 1:** Chemical shift assignment of human Hsp90.

Domain (+ligand)/Construct	Assignment			Reference
	Resonances—Backbone/Methyl Groups	% of Assigned Residues	% of Unassigned Residues	
NTD	^1^H, ^15^N, ^13^C—backbone	82% of backbone and C_β_ resonances (9–223) 89% of C_α_, 80% of C_β_ (17–224) 81% of NH ^1^	18% of backbone and C_β_ resonances (9–223) 11% of C_α_, 20% of C_β_ (17–224) 19% of NH ^1^	BMRB: 7003, Jacobs et al., 2006 [41] BMRB: 19,560, Park et al., 2011 [14] BMRB: 50,786, Henot et al., 2021 [40]
	^1^H, ^13^C—methyl groups AIL^pro-S^MTV^pro-S^	100% of AIL^pro-S^MTV^pro-S^	0% of AIL^pro-S^MTV^pro-S^	BMRB: 19,560, Park et al., 2011 [14] Karagöz et al., 2011 [9] BMRB: 50,786, Henot et al., 2021 [40]
NTD + ligand	^1^H, ^15^N, ^13^C—backbone	96% of C_α_, 95% of C_β_ (non-proline residues) 92% of non-proline backbone (NH) 85% of NH ^1^	8% of non-proline backbone (NH) 4% of C_α_, 5% of C_β_ (non-proline residues) 15% of NH ^1^	BMRB: 51,378, Henot et al., 2022 [39]
	^1^H, ^13^C—methyl groups AIL^pro-S^MTV^pro-S^	100% of AIL^pro-S^MTV^pro-S^	0% of AIL^pro-S^MTV^pro-S^	
MD	^1^H, ^15^N, ^13^C—backbone ^1^H, ^13^C—methyl groups Ile-δ_1_	82% of NH ^1^ 100% of all Ile-δ_1_	18% of NH ^1^ 0% of all Ile-δ_1_	BMRB: 19,560, Park et al., 2011 [14] Karagöz et al., 2011 [9]
NTD + MD	^1^H, ^15^N—backbone ^1^H, ^13^C—methyl groups Ile-δ_1_	/ ^2^ 95% of all Ile-δ_1_	/ ^2^ 5% of all Ile-δ_1_	BMRB: 19,560, Park et al., 2011 [14]
Hsp90Δ	^1^H, ^13^C—methyl groups Ile-δ_1_	/ ^2^	/ ^2^	BMRB: 19,560, Park et al., 2011 [14]
full-length	^1^H, ^13^C—methyl groups Ile-δ_1_ and Met-ε	100% of all Ile-δ_1_ 35% of all Met-ε	0% of all Ile-δ_1_ 65% of all Met-ε	Karagöz et al., 2011 [9] Oroz et al., 2017–2019 [20,38,42] Lopez et al., 2021 [22]

^1^ according to the biological magnetic resonance bank (BMRB). ^2^ Park et al. (2011) did not indicate the % of assigned residues [14].

**Table 2:** Perturbed Ile-δ_1_ resonances in methyl transverse relaxation-optimized spectroscopy (TROSY) spectra after the binding of ATP or its analogs, p23 in the presence of ATP or its analogs (ATP + p23), adenosine diphosphate (ADP), the activator of Hsp90 ATPase protein 1 (Aha1) or the Hsp70/Hsp90-organizing protein (Hop) to full-length Hsp90β with specifically ^13^CH_3_-labeled Ile-δ_1_ methyl groups (marked by X).

		ATP	ATP + p23	ADP	Aha1	Hop
**NTD**	I20	X ^1^	X	X	X ^1^	X ^1^
	I27	X ^1^	X	X	X ^1^	
	I28	X ^1^	X	X	X	
	I37			X	X ^1^	
	I53	X				
	I75	X			X ^1^	X ^1^
	I90	X	X			
	I98			X	X	
	I122	X	X	X	X ^2^	
	I125			X	X ^2^	
	I208				X ^1^	
**MD**	I287					X
	I334					X
	I361			X	X	
	I369		X ^1^			
	I376					X
	I389			X	X	
	I399		X ^2^			
	I407					X
	I440		X ^1^			
	I482		X ^1^			
	I485					X
**CTD**	I590					X
	I604					X
	I679					X

^1^ conformational changes, ^2^ interaction in the process of binding.

## Data Availability

Data sharing is not applicable.

## Abbreviations

AMP-PNP, adenylyl imidodiphosphate; ANP, phosphoaminophosphonic acid-adenylate ester; ATP, adenosine triphosphate; Aha1, activator of Hsp90 ATPase protein 1; ADP, adenosine diphosphate; BMRB, biological magnetic resonance bank; CEST, chemical exchange saturation transfer; CPMG, Carr–Purcell–Meiboom–Gill; CRINEPT, cross-relaxation enhanced polarization transfer; cryo-EM, cryogenic electron microscopy; CSP, chemical shift perturbation; CTD, C-terminal domain; CTI, synthetic small-molecule allosteric modulator targeting the CTD; Erα, estrogen receptor α; GEM, group epitope mapping; GR, glucocorticoid receptor; Hop, Hsp70/Hsp90-organizing protein; Hsp90, heat shock protein 90; HSR, heat shock response; HSQC, heteronuclear single quantum coherence; ITC, isothermal titration calorimetry; MD, middle domain; MR, mineralocorticoid receptor; NMR, nuclear magnetic resonance; NOE, nuclear Overhauser effect; trNOESY, transferred nuclear Overhauser effect spectroscopy; NTD, N-terminal domain; NTI, N-terminal inhibitor; PDB, protein data bank; PK/LDH, pyruvate kinase/lactic dehydrogenase; PRE, paramagnetic relaxation enhancement; c-Raf, c-rapidly accelerated fibrosarcoma; SEC-MALS, size-exclusion chromatography with multi-angle static light scattering; TNBC, triple negative breast cancer; TR-FRET, time-resolved fluorescence energy transfer; TROSY, transverse relaxation-optimized spectroscopy; wt, wild type
